# A rare case of *Curtobacterium flaccumfaciens* infection in the eye: a case report

**DOI:** 10.1186/s41182-022-00452-1

**Published:** 2022-09-05

**Authors:** Pauline Mallick

**Affiliations:** Cairns Base Hospital, 165 The Esplanade, Cairns City, QLD 4870 Australia

**Keywords:** *Curtobacterium flaccumfaciens*, Infective keratitis, Eye

## Abstract

**Purpose:**

To report a rare case of microbial keratitis caused by *Curtobacterium flaccumfaciens (C. flaccumfaciens*) infection.

**Methods and results:**

A 24-year-old male presented with pain and redness of the eye following ocular exposure to banana bell sap. He was diagnosed with infectious keratitis and underwent corneal scraping. Subsequent culture and molecular analysis identified the causative organism as *C. flaccumfaciens.* Keratitis clinically resolved following several weeks of topical therapy with ciprofloxacin 0.3%.

**Conclusion:**

*Curtobacterium flaccumfaciens* is a rare and likely underdiagnosed cause of bacterial keratitis. This report provides further evidence of its pathogenic role in not only plants, but also the human cornea, especially in the tropics.

## Introduction

Bacterial keratitis is a commonly encountered ophthalmic pathology and a major cause of corneal blindness. Common bacterial pathogens include *Staphylococcus* spp., *Streptococcus* spp., and *Pseudomonas* spp., which have higher prevalence rates in tropical climates [1, 2].

A case of bacterial keratitis caused by *Curtobacterium flaccumfaciens* was reported in a Vanuatuan man after accidentally getting banana bell sap in his right eye. This case report explores how *C. flaccumfaciens* may be underdiagnosed due to laboratory conditions preventing optimal growth, making it difficult for microbiologists to isolate this pathogen from clinical samples. In this case, the organism was grown from corneal scrape samples for > 24 h after collection.

## Case report

A previously healthy 24-year-old Vanuatuan man presented with redness and pain in his right eye one day after ocular exposure to white sap from a banana bell. This occurred while working in the Atherton Tablelands, Queensland. He denied any direct ocular contact with the branches, leaves, or soil and was not a contact lens wearer. He presented with irritation and photophobia in his right eye, associated with excessive lacrimation; he was otherwise systemically healthy. He had no previous ocular or general medical history. He had no known allergies, including latex allergy.

On examination, his visual acuity was 20/16 in both eyes. Slit lamp examination of his right eye revealed diffuse conjunctival injection and an inferior midperipheral corneal defect measuring 1.5 cm × 1.5 cm with underlying faint white anterior stromal infiltrate. He had mild anterior chamber activity (0.5 + cells). The dilated fundus examination was unremarkable.

Corneal scraping was performed, and samples were sent for microscopy, culture, and sensitivity testing. Under aerobic conditions at 35 °C in 5% CO_2_, growth of *C. flaccumfaciens* was observed on chocolate agar plates at 48 h. Along with biochemical typing, the isolate was subjected to mass spectrometry fingerprinting with matrix-assisted laser desorption ionization-time of flight mass spectrometry (MALDI-ToF MS). Gram staining showed Gram-positive bacilli, and the colonies were motile, nonhemolytic and smooth.

**Fig. 1:**
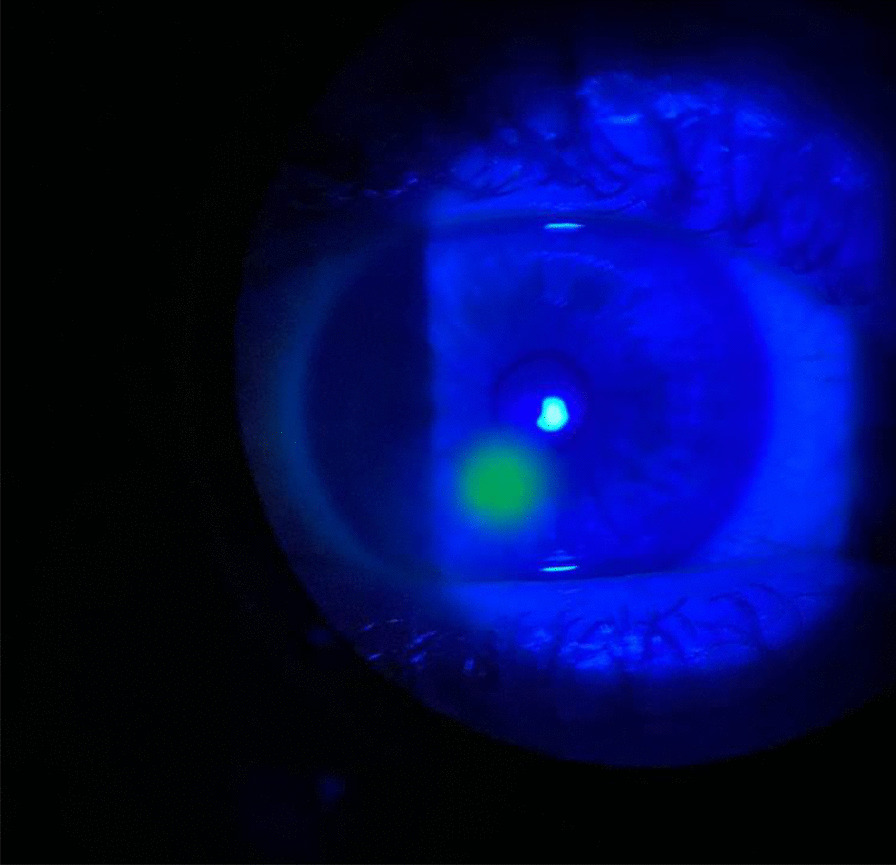
Anterior segment slit lamp examination showing an area of homogenous fluorescein uptake located inferiorly

The main differential diagnosis was a chemical burn from the banana sap, given its homogenous fluorescein uptake, as seen in Fig. 1. Empirical antibiotic treatment was started in this patient since the underlying anterior stromal infiltrate was more suggestive of microbial keratitis than chemical burn. The patient started monotherapy with ciprofloxacin 0.3% eye drops every hour for the first 24 h and then every 2 h for the next 24 h. At the 48-h follow-up, microbial keratitis was more likely, as the lesion had responded to treatment, with both infiltrate and epithelial defects decreasing in size. Topical ciprofloxacin administration was reduced to only waking hours and slowly weaned to 1 drop every week over the course of 5 weeks, with gradual clinical resolution. Visual acuity remained unchanged throughout. A final follow-up was performed over the phone due to coronavirus disease 2019 (COVID-19) restrictions, which prevented reexamination of the eye, but subjectively, the patient noted substantial improvement in his ophthalmic symptoms with no further pain or sensitivity. He received a total of 5 weeks of treatment, and ongoing advice was given by the optometrist 2 weeks after the phone follow-up.

## Discussion

*Curtobacterium* *flaccumfaciens *is a weakly Gram-positive, motile rod-like organism belonging to the order Micrococcales [3]. *C. flaccumfaciens* is one of eight strains belonging to the genus *Curtobacterium*; it is the least studied coryneform bacterium. The pathogenicity of *C. flaccumfaciens* is considered low, and it is typically regarded as a plant pathogen; recorded plant cases involved crops and ornamental plants, such as field beans, beets, tulip bulbs and poinsettia, typically causing wilting and brown spots [4–6]. Due to its ubiquity in vegetable matter, no connection has been formally described between this organism and banana trees. This report of infective keratitis due to banana sap exposure is the first well-documented ocular presentation of *C. flaccumfaciens* infection in humans. To date, *Curtobacterium* has been isolated from only eye discharge in a 34-year-old female with conjunctivitis; however, the pathogenicity was not thoroughly discussed.

The only other well-documented case of human infection with this organism was in a child with septic arthritis; the causative agent was first phenotypically isolated by Gram staining and then further identified using a MicroSEQ500 16S rDNA bacterial identification kit in Foster City, CA [3]. Molecular identification analysis according to 467 nucleotides was performed, and the bacterium demonstrated 99.89% sequence homology with *C. flaccumfaciens.* Similar to this case, yellow-pigmented Gram-positive bacilli were isolated from the synovial tissue of the child, the only difference being that this strain was nonmotile.

Multiple reports have noted that clinical laboratory growth of this pathogen is challenging, as optimal growth occurs at 30 °C; however, plates are routinely incubated at 35–37 °C. This may mean that some strains may not be isolated from specimens [4, 5]. In addition, the growth of *C. flaccumfaciens* on complex media is slow, requiring 3 days for visible colony formation [3, 6]. This slow growth further contributes to underdiagnosis in clinical settings and may explain the paucity of documented cases of human infection despite the probable high rates of exposure in agricultural settings. Additionally, detection of this pathogen may be limited due to its lack of inclusion in databases of commercial identification systems, as they are not commonly encountered coryneform bacteria. [3]

This pathogen was preliminarily identified as a Gram-positive bacillus on Gram staining, and the colonies were yellowish, motile, nonhemolytic, and smooth, similar to those reported by Funke et al. [4]. The colonies grew on horse blood agar and chocolate agar at 48 h under aerobic conditions at 35 °C at Cairns Hospital laboratory. MALDI-ToF MS was performed on one colony that was first isolated on the chocolate agar plate, and α-cyano-4-hydroxycinnamic acid (HCCA) matrix substance was prepared with the isolate. *C. flaccumfaciens* was identified by comparing its peptide mass fingerprint (PMF) to the PMFs in the database. The microbe was further prepared in formic acid, which studies have shown improves the diagnostic potential of MALDI-ToF MS [7, 8]. It demonstrated 50.1% sequence homology with *C. flaccumfaciens* and 49.9% homology with *Curtobacterium ammoniigenes (C. ammoniigenes)*. Typical phenotypic characteristics of *C. flaccumfaciens* include yellow- or orange-pigmentation, oxidase positive, motility, catalase positivity, nitrate reductase negativity, urease negativity, and strongly hydrolyzed esculin [4].

In this case, culture revealed sensitivity to ciprofloxacin, with a zone of inhibition size of 27 mm. This was further confirmed by the rapid clinical response to monotherapy. The pathogen was also sensitive to vancomycin (25 mm), tetracycline (28 mm), daptomycin (31 mm) and gentamicin (29 mm). The strain was resistant to penicillin. Antimicrobial susceptibilities of 15 *Curtobacterium* isolates were reported in Funke et al., with the 50% and 90% minimum inhibitory concentrations (MICs) of beta-lactams being greater than 1 μg/ml. The MICs for macrolides (except azithromycin) and rifampicin were ≤ 0.03 μg/ml for all strains evaluated. The MICs of vancomycin were lower than 2 μg/ml for all strains examined [4]. These findings suggest that *C. flaccumfaciens* may be effectively treated with multiple common ophthalmic antibiotic agents. As such, infection with this organism is associated with a good prognosis, defined as the complete resolution of ocular symptoms, as was observed in this case. If left untreated, however, microbial keratitis could result in chronic corneal inflammation and scarring, scleral extension of infection, irregular astigmatism, a permanent reduction in vision, corneal perforation, endophthalmitis, and blindness.

The level of conclusiveness of phenotypic identification could have been limited by the fact that no sensitivities were reported and that *C. Ammoniigenes* was also isolated. To evaluate sensitivity, calibrations must be performed; however, because this pathogen is rarely isolated from the environment, the CBH laboratory was unable to set specific sensitivity zones. Another limitation was the interlaboratory reproducibility of PMFs, as PMFs are dependent on the quality of the samples and affected by the sample preparation technique.

## Conclusion

This report of infective keratitis from banana sap is the second most well-documented clinical presentation of *C. flaccumfaciens* infection in a person. This case report, along with the septic arthritis case report, can increase awareness of the pathogen among workers in tropical areas. Its detection is limited by diagnostic capabilities, but current molecular techniques in clinical laboratories, such as MicroSEQ500 16S rDNA bacterial identification and MALDI-ToF MS, will help identify additional cases in people. MALDI-ToF MS is a rapid, reliable and cost-effective method for the identification of organisms in clinical samples to ensure appropriate antimicrobial therapy is started. This system allows the identification of rare isolates, such as *C. flaccumfaciens*, by comparing their PMFs to reference PMFs in the database. This highlights the importance of maintaining an extensive database containing reference PMFs of multiple strains of a variety of species.

Awareness of different pathogens causing infectious keratitis is important for ensuring appropriate antibiotic therapy to prevent vision impairment and reduce patient morbidity. This is especially important in rural tropical regions and developing nations, where many young laborers harvest fruit. Often, workers do not wear appropriate eyewear and equipment to protect them from pathogens endemic to the tropics. Since *C. flaccumfaciens* has multiple common ophthalmic antibiotic sensitivities, empiric treatment with a sensitive antibiotic should be started if clinical suspicion for microbial keratitis is high.

Documentation of these cases of human infection in clinical literature may aid in the discovery of more strains belonging to this genus in clinical specimens and the development of further investigative techniques. Through this research and awareness of different pathogens, clinicians can better target medical treatment to prevent increased mortality and potentially sight-threatening effects due to infective keratitis.

## Data Availability

Not applicable.
